# Vitamin D Supplementation Has No Impact on Cardiorespiratory Fitness, but Improves Inflammatory Status in Vitamin D Deficient Young Men Engaged in Resistance Training

**DOI:** 10.3390/nu14245302

**Published:** 2022-12-13

**Authors:** Lauri Savolainen, Saima Timpmann, Martin Mooses, Luule Medijainen, Lisette Tõnutare, Frederik Ross, Märt Lellsaar, Anneli Piir, Mihkel Zilmer, Eve Unt, Vahur Ööpik

**Affiliations:** 1 Institute of Sport Sciences and Physiotherapy, University of Tartu, 18 Ülikooli St., 50090 Tartu, Estonia; 2 Department of Biochemistry, Institute of Biomedicine and Translational Medicine, University of Tartu, 50090 Tartu, Estonia; 3 Department of Cardiology, Institute of Clinical Medicine, University of Tartu, 50090 Tartu, Estonia; 4 Department of Sport Medicine and Rehabilitation, Institute of Clinical Medicine, University of Tartu, 50090 Tartu, Estonia; 5 Sport Medicine and Rehabilitation Clinic, Tartu University Hospital, 1a Puusepa St., 50406 Tartu, Estonia

**Keywords:** vitamin D, cytokines, low-grade inflammation, maximal oxygen consumption

## Abstract

Data on the effect of vitamin D (Vit-D) supplementation on cardiorespiratory fitness (VO_2_max) are conflicting. A possible source of discrepancies in the literature is the heterogeneity in baseline Vit-D status among participants in previous studies. The main objectives of the present study were to assess the impact of Vit-D supplementation on VO_2_max and inflammatory status in Vit-D deficient young healthy men. Participants (n = 39, baseline serum Vit-D level < 50 nmol/L) were quasi-randomly assigned to one of the two groups, which, in a double-blind manner, supplemented their diet daily with either Vit-D (8000 IU; VD) or placebo (PLC) and concomitantly performed a 12-week supervised resistance training program. During the 12-week intervention, serum Vit-D concentrations increased 3.9-fold (*p* < 0.001) in the VD group while no changes occurred in the PLC group. Baseline VO_2_max did not differ in the two groups and remained unchanged during the intervention. Serum interleukin-10/tumour necrosis factor alpha ratio increased significantly (30%, *p* = 0.007; effect size 0.399) in VD but not in PLC group. In conclusion, 12-week Vit-D supplementation increases serum 25(OH)D levels and improves inflammatory status, but has no impact on VO_2_max in Vit-D deficient young men engaged in resistance training.

## 1. Introduction

Vitamin D (Vit-D) is a fat-soluble vitamin, the bioactive form of which (calcitriol) acts in the human body like a steroid hormone, i.e., through specific nuclear receptors [1,2,3]. Vit-D receptors (VDR) have been detected in most cells and tissues in the human body [4,5,6], including skeletal muscle [7,8], vascular smooth muscle [9], lungs [10], cardiac muscle [11], and immune cells [12].

Maximal oxygen uptake (VO_2_max)—the maximal rate of oxygen consumption by the body during maximal dynamic exercise involving large muscle mass—is considered the most valid measure of functional capacity of the cardiorespiratory system and is often referred to as cardiorespiratory fitness [13]. The major factors determining an individual’s VO_2_max are the functional state of the lungs, heart, vasculature, and skeletal muscle as well as the oxygen transport capacity of the blood [14]. Given that VDR is expressed in all of these organs (lungs, heart, blood vessels, skeletal muscle), it is reasonable to assume that Vit-D may affect their function and thus VO_2_max. Furthermore, Vit-D may promote erythropoiesis and haemoglobin synthesis [15] and affect the binding affinity of oxygen to haemoglobin [16]. It is therefore not surprising that Ardestani et al. [17] and Marawan et al. [18] reported an independent robust association between serum Vit-D levels and VO_2_max in adults over a wide age range. However, studies in adolescents did not show such an association [19], or showed that it occurred only in boys but not in girls [20].

On the other hand, many experimental studies concluded that Vit-D supplementation has no impact on VO_2_max in subjects with varying age, training status, and baseline serum Vit-D levels [21,22,23,24,25,26]. Nevertheless, some recent publications reported a positive effect [27,28,29].

It is well established that cardiorespiratory fitness, measured as VO_2_max, is inversely associated with cardiovascular disease risk and all-cause mortality [30,31]. Chronic low-grade inflammation, on the other hand, plays a critical role in the pathogenesis of atherosclerosis, the latter being the main cause of cardiovascular diseases [32,33,34]. Calcitriol, the bioactive form of Vit-D, inhibits and stimulates the production of pro- and anti-inflammatory cytokines, respectively, [3,35,36] and Vit-D supplementation in healthy adults, depending on dose, has analogous impact on cytokine production in monocyte-derived macrophages [37]. Different Vit-D supplementation regimens [38,39,40], resistance training (RT) [41,42], or Vit-D supplementation in combination with RT [39,43] show anti-inflammatory effects in patients suffering under various diseases characterized by high level chronic inflammation. According to Forti et al. [44] and Ihalainen et al. [45], suppression of chronic inflammation at a young age can be an effective measure to prevent or delay the onset of inflammation and related diseases later in life, and they have demonstrated anti-inflammatory effects of various RT programs in young healthy adults. These researchers [44,45] did not control Vit-D status of their subjects and to the best of our knowledge, the potential additive effect of Vit-D supplementation on anti-inflammatory responses to RT in Vit-D deficient young adults has not been studied at all.

Thus, the data regarding the impact of Vit-D supplementation on cardiorespiratory fitness in subjects with varying Vit-D status is controversial and there is a lack of knowledge on the potential additive effect of Vit-D supplementation on anti-inflammatory responses to RT in Vit-D deficient young men. Therefore, the aim of the current study was to test the hypothesis according to which Vit-D supplementation in Vit-D deficient young men would have a positive effect on VO_2_max and potentiate the anti-inflammatory effect of RT. Our hypothesis is based on the assumption that these effects of Vit-D supplementation, if present, are more likely to occur in subjects with Vit-D deficiency than in individuals with normal Vit-D status.

## 2. Materials and Methods

### 2.1. Study Design and Participants

The present work is based on unpublished data collected in a double-blind placebo controlled quasi-randomized study [46] which assessed the effects of high dose Vit-D supplementation on muscle strength and body composition in Vit-D deficient young men participating in a supervised RT program.

Young male volunteers were invited to the study through various university mailing lists. The initial inclusion criteria required subjects to be without chronic diseases, not using Vit-D supplements, and not participating in competitive sports or in any recreational RT program during the last year. The number of volunteers who met the initial inclusion criteria was 60. All these men were invited to face-to-face meeting where they received detailed information regarding the aims, research procedures and duration of the study. They were also informed that volunteers with a baseline serum 25(OH)D level ≥ 60 nmol/L and those with a serum level ≥ 50 nmol/L a month after baseline measurement will be excluded from the further study. After signing informed consent, volunteers donated baseline venous blood sample. Eight men whose baseline serum 25(OH)D level exceeded 60 nmol/L were excluded from the rest of the study whereas the other 52 men entered the 4-week preparatory phase of the research (see the next section). During the preparatory phase, 8 men dropped out of the study due to lack of time and/or motivation. Another 3 men were excluded at the end of the preparatory phase as their serum 25(OH)D level exceeded the 50 nmol/L criterion. The remaining 41 Vit-D deficient participants were quasi-randomly divided into Vit-D supplemented (VD) and placebo (PLC) groups for the 12-week main phase of the study (see the next section). Quasi-randomization means that participants were ranked in ascending order of their body weight and then assigned to alternative groups. The 41 participants who entered the main phase of the study were considered Vit-D deficient based on their serum 25(OH)D levels of less than 50 nmol/L [47,48]. During the 12-week main phase, one participant dropped out of the study due to shortage of time and one more because of loss of motivation. Thus, there were 39 men who participated in at least 80% of the training sessions, donated all necessary blood samples, and whose data were included in the final analysis. Their age, height, body mass, and body mass index were (mean ± SD) 23.7 ± 2.5 years, 1.83 ± 0.6 m, 79.7 ± 9.7 kg, and 23.7 ± 2.5 kg/m^2^, respectively, at the beginning of the preparatory phase of the study.

### 2.2. General Organization of the Study

After recruitment process in October and November (see the preceding section), the whole 16-week study was carried out from December to March. The prevalence of Vit-D deficiency among young Estonian men is very high during these months—the winter-spring period [49].

In the preparatory phase (the first 4 weeks), participants were taught to use RT machines and to perform the exercises technically correctly. They were also taught to keep a 4-day food diary and enter nutritional information into the online Nutridata platform (National Institute for Health Development, Estonia).

In the main phase (the next 12 weeks), the participants in both VD and PLC groups trained regularly for 3 days a week and simultaneously administered Vit-D (VD group) or placebo (PLC group). In the second, sixth, and eleventh weeks of the main phase, participants completed 4-day food diaries in the Nutridata online platform. Participants gave access to their diaries to a member of the research team for data analysis.

Serum 25(OH)D data were collected in 4-week intervals beginning from the recruitment process until the end of the main phase of the study, but serum interleukin levels were assessed before and after the 12-week main phase only.

### 2.3. Resistance Training and Dietary Supplementation

The training program consisted of 7 exercises [50] on RT machines and involved both upper and lower body muscles. Training loads were gradually increased according to improvements in participants’ performance during the 12-week training period. Detailed description of the training program and dosing of the training loads is given in Savolainen et al. [46].

From the beginning to the end of the 12-week training period, participants in the VD and PLC groups administered vitamin D_3_ and placebo, respectively, daily in a double-blind manner. The vitamin D_3_ and placebo supplements (product codes ST45851 and ST47202, respectively; Diafarm A/S, Vejle, Denmark) were packed in gelatine capsules and could not be distinguished from each other. The daily dose of vitamin D_3_ administered to the participants in the VD group was 8000 IU. According to Pludowski et al. [51], the minimal serum 25(OH)D level required for triggering extra-skeletal effects of Vit-D is approximately 75–125 nmol/L. Thus, using a high daily dose of supplemental Vit-D, our goal was to increase serum 25(OH)D levels above 125 nmol/L in the VD group.

On training days only, within approximately 30 min after workout, participants in both groups ingested 25 g of whey-based supplement (Whey 80, Elite Fitness OY, Helsinki, Finland). This measure was used to standardize the potential impact of protein intake on the early recovery processes that may affect the outcomes of a several-week RT program [52].

### 2.4. Measurement of Maximal Oxygen Uptake

Participants underwent a graded maximal exercise test to determine the highest level of oxygen uptake using a breath-by-breath metabolic system (MasterScreen CPX, Viasys Healthcare GmbH, Hoechberg, Germany) and a motorized treadmill (Viasys/Jaeger LE300 C, Viasys Healthcare GmbH, Hoechberg, Germany) for two times: before and after the 12-week supplementation/RT period. An incremental running test was employed that started with the speed of the treadmill belt at 6.0 km/h and an incline of 1.5%. The incline remained constant, but the speed of the treadmill belt was increased by 2 km/h every 3 min until self-determined exhaustion. Breathing gas was analysed automatically for every 5-s period. VO_2_max was considered as the highest mean rate of oxygen uptake achieved within 30 s at the end of the test. Criteria for achieving VO_2_max included respiratory exchange ratio (RER) > 1.00 and heart rate (HR) > 90% of the participant’s age-predicted maximum [53]. Heart rate was recorded continuously at 5-s intervals during the running test by means of a sport-tester (Polar Electro, Finland).

### 2.5. Blood Sampling and Analyses

Venous blood samples were drawn in the morning after two days of rest, approximately 12 h after the last meal. Blood was collected into 5 mL Vacutainer serum tubes as well as into 3 mL Vacutainer tubes containing EDTA. The blood from EDTA tube was used for the measurement of haemogram using analyser Sysmex XE-5000 (Sysmex Corporation, Kobe, Japan). Blood collected into serum tubes was allowed to clot, and then the serum was separated by centrifugation as described in Savolainen et al. [46]. After centrifugation, the tubes were still maintained at 4 °C and transported to the United Laboratories of the Tartu University Hospital for analysis.

Serum 25(OH)D concentration was measured by chemiluminescence immunoassay method using IDS-iSYS Multi-Discipline Automated Analyser (Immunodiagnostic Systems Limited, Copenhagen, Denmark). Serum glucose concentration was measured using the hexokinase enzymatic method on Roche/Hitachi Cobas 6000 c501 analyser (Roche Diagnostics GmbH, Mannheim, Germany). Concentration of insulin was measured by electrochemiluminescence immunoassay “ECLIA” on Cobas 6000 analyser (Roche Diagnostics GmbH, Tokyo, Japan). All these analyses were performed in the United Laboratories of the Tartu University Hospital. At the Institute of Biomedicine and Translational Medicine of the University of Tartu, the Evidence Investigator Cytokine and Growth Factors High-Sensitivity Array based on the sandwich chemiluminescent immunoassay, version V1.4.1 (RANDOX Laboratories Ltd., Crumlin, UK) was used for simultaneous quantitative detection of cytokines. The concentrations of the following proteins were measured from a single sample: interleukin 1α (IL-1α), interleukin 1β (IL-1β), interleukin 4 (IL-4), interleukin 6 (IL-6), interleukin 8 (IL-8), interleukin 10 (IL-10), tumour necrosis factor alpha (TNF-α) and monocyte chemoattractant protein 1 (MCP-1). The core technology Randox Biochip contains an array of discrete test regions of immobilized antibodies specific to different cytokines and growth factors.

The HOMA-IR index (homeostasis model of assessment of insulin resistance) was calculated as HOMA-IR = (serum glucose (mmol/L) × serum insulin (μU/mL))/22.5 [54].

### 2.6. Statistical Analysis

Data were analysed using Statistica 13.3 software (TIBCO Software Inc., Palo Alto, CA, USA) and presented as means ± SD. All data were checked for normal distribution using the Kolmogorov–Smirnov test, which revealed that IL-1α, IL-1β, and IL-6 were not normally distributed and were therefore log-transformed. A two-way repeated analysis of variance ANOVA with a between factor of group (VD vs. PLC) and within factor of time was used to evaluate the differences within and between the groups. If a significant main effect or interaction occurred, Tukey’s honestly significant difference post hoc analysis was used to locate differences between the means. Effect size as Cohen’s *d* [55] was calculated for IL-10/TNF-α ratio in the VD group. The mean values of different parameters registered at a single time point were compared using Student’s *t* test for independent variables. Significance was set at *p* < 0.05 level.

## 3. Results

**Figure 1:**
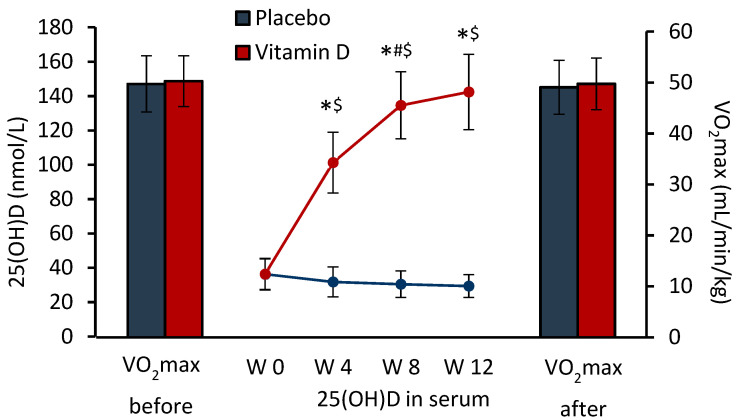
Serum 25(OH)D concentration (lines) and VO_2_max (columns) during 12-week supplementation and resistance training period. Data are presented as mean ± SD; n = 17 in placebo and n = 20 in vitamin D group. W 0—W 12, weeks 0—12. Significantly different (*p* < 0.05): * from W 0; # from previous week; $ from placebo group.

Serum 25(OH)D levels increased (3.9-fold on average) in the VD group from week 0 to week 12, while there was a statistically non-significant (19% on average) decrease in the PLC group (main effects of time and group: in both cases *p* < 0.001; group-by-time interaction: *p* < 0.001; Figure 1). VO_2_max did not differ in the two groups and remained unchanged during the 12-week supplementation and RT period (main effects of group and time: *p* = 0.716 and *p* = 0.200, respectively; group-by-time interaction: *p* = 0.890; Figure 1).

There were no significant main effects of group (*p* = 0.990) or time (*p* = 0.159) for RER (Table 1). However, small but statistically significant increase in RER appeared in the VD group across the 12-week supplementation and RT period (group-by-time interaction: *p* = 0.027; Table 1). Peak HR registered during VO_2_max test did not differ in the two groups (main effect of group: *p* = 0.546) and did not change during the 12-week supplementation and RT period (main effect of time: *p* = 0.257; group-by-time interaction: *p* = 0.580). No significant main effects of group or time, or group-by-time interactions appeared for VE or BF levels (in all cases *p* > 0.05; Table 1).

No significant main effects of group or time, or group-by-time interactions appeared for serum IL-1α, IL-1β, IL-4, IL-6, IL-8, TNF-α, and MCP-1 levels (in all cases *p* > 0.05; Table 2). A significant main effect of time (*p* = 0.031) without significant main effect of group (*p* = 0.601) or group-by-time interaction (*p* = 0.352) occurred for IL-10. Overall serum IL-10 levels increased by an average 17.1% from 0.70 ± 0.41 pg/mL at week 0 to 0.82 ± 0.51 pg/mL at week 12 (*p* = 0.026). A significant main effect of time (*p* = 0.003) but not of group (*p* = 0.137) or group-by-time interaction (*p* = 0.134) occurred for IL-10/TNF-α ratio. However, there was a significant increase in IL-10/TNF-α ratio in the VD group (*p* = 0.007, Cohen’s *d* = 0.399) but not in the PLC group (*p* = 0.671) across the 12-week supplementation and RT period (Table 2).

There was a significant main effect of group (*p* = 0.023), but no significant main effect of time (*p* = 0.068) or group-by-time interaction (*p* = 0.248) for serum glucose levels (Table 3). Overall serum glucose concentration in the PLC group (5.36 ± 0.31 mmol/L) exceeded that observed in the VD group (5.14 ± 0.38 mmol/L) by an average 4.3% (*p* = 0.023).

Similarly, there was a significant main effect of group (*p* = 0.025) with no significant main effect of time (*p* = 0.637) or group-by-time interaction (*p* = 0.912) for serum insulin levels (Table 3). Overall serum insulin levels in the PLC group (9.28 ± 2.83 mU/L) were on average 19.4% higher than in the VD group (7.77 ± 2.65 mU/L; *p* = 0.025).

There was a significant main effect of group (*p* = 0.013), but no significant main effect of time (*p* = 0.890) or group-by-time interaction (*p* = 0.774) for HOMA-IR (Table 3). Overall HOMA-IR was higher in the PLC group compared to the VD group (2.22 ± 0.74 and 1.78 ± 0.66, respectively; *p* = 0.013).

A significant main effect of time (*p* < 0.001), but not that of group (*p* = 0.249) occurred for serum ferritin levels (Table 3). There was no group-by-time interaction (*p* = 0.936) for serum ferritin levels. Overall serum ferritin levels decreased from 117.3 ± 77.3 µg/L at week 0 to 91.0 ± 72.2 µg/L at week 12, i.e., by an average 22.4% (*p* < 0.001). The number of participants with serum ferritin levels ≤35 µg/L was six (4 in PLC and 2 in VD group) at week 0, and eight at week 12 (5 in PLC and 3 in VD group).

No significant between-group differences or changes over time occurred in haemoglobin, haematocrit, or blood cell count (main effects of group and time, and group-by-time interaction: in all cases *p* > 0.05; Table 4).

## 4. Discussion

In the present study, 12-week daily Vit-D supplementation increased the mean serum 25(OH)D concentration to the level of 142.4 nmol/L in initially Vit-D deficient young healthy men. However, contrary to our hypothesis, the remarkable positive shift in Vit-D status had no impact on VO_2_max, which remained unchanged during the supplementation and RT period.

This finding is in line with the data of others who have reported no effect of Vit-D supplementation on VO_2_max in young and middle-aged overweight and obese adults [21,22], healthy male adults [25], healthy male and female Gaelic footballers [23], male elite soccer players [24], and young male military recruits [26]. However, most of these studies [21,22,23,24,25] included, in varying proportions, both individuals with Vit-D deficiency and subjects with sufficient baseline Vit-D status. On the other hand, some of the effects of Vit-D supplementation, such as on muscle [56] and immune function [57], are more pronounced in individuals with low baseline serum 25(OH)D levels. Thus, the ability of most previous studies [21,22,23,24,25] to detect the potential effect of Vit-D supplementation on VO_2_max may have been limited due to the inclusion of both Vit-D deficient and sufficient subjects.

Nevertheless, Menon et al. [26] studied exclusively Vit-D deficient individuals like ours and similarly to us reported no effect of Vit-D supplementation on VO_2_max. However, the reliability of their conclusion is weak because these researchers did not measure but indirectly calculated VO_2_max. The VO_2_max levels reported by Menon et al. [26] exceeded 76 and 86 mL/min/kg before and after 12-week supplementation period, respectively, in both Vit-D and placebo supplemented groups. These values are unrealistically high for ordinary military recruits as they clearly surpass the VO_2_max levels measured in elite endurance athletes [58]. The ability of Menon et al. [26] to objectively evaluate the potential impact of Vit-D supplementation on VO_2_max was further compromised by the fact that serum 25(OH)D levels increased significantly in both Vit-D supplemented and control (non-supplemented) groups, meaning that all their participants were in Vit-D sufficient status after the supplementation period.

Contrary to our findings, Ramezani Ahmadi et al. [28] reported higher post-supplementation VO_2_max levels in Vit-D supplemented male physical education students than in their placebo-supplemented counterparts. Similarly, Jastrzębska et al. [27] and Kujach et al. [29] observed greater positive changes in VO_2_max across supplementation period in Vit-D compared to placebo supplemented well-trained young soccer players and healthy physically active males, respectively. However, Ramezani Ahmadi et al. [28] and Jastrzębska et al. [27] calculated VO_2_max levels indirectly, while Kujach et al. [29] did not actually observe an increase in VO_2_max in the Vit-D supplemented group, but recorded a larger decrease in this parameter in the placebo group. Furthermore, serum 25(OH)D levels in the Vit-D supplemented participants studied by Ramezani Ahmadi et al. [28] did not change across supplementation period. Given these limitations, the results of the three studies [27,28,29] do not provide reliable evidence to support a causal relationship between Vit-D supplementation and VO_2_max improvement.

Thus, given the limitations of the previous studies discussed above, our data are the strongest evidence to date that Vit-D supplementation does not improve VO_2_max in Vit-D deficient young healthy men. We consider it unlikely that, in our participants, involvement in RT program and/or protein supplementation on training days suppressed the potential impact of Vit-D on VO_2_max, because both RT [59] and protein supplementation [60] can have positive rather than negative effect on VO_2_max. In fact, in the present study, neither RT with protein supplementation (PLC group) nor RT with protein and Vit-D supplementation (VD group) induced changes in VO_2_max.

Some observational studies [61,62] have revealed positive association between Vit-D status and blood haemoglobin concentration, and according to Smith and Tangpricha [15] Vit-D may promote the synthesis of haemoglobin and erythropoiesis. In addition, Kujach et al. [29] reported increases in maximal breath frequency and maximal lung ventilation due to Vit-D supplementation. Both increased haemoglobin levels and improved lung function may favour increases in VO_2_max. However, in our participants, no impact of Vit-D supplementation and/or 12-week RT on blood haemoglobin levels, any other haematological parameter, maximal breath frequency or maximal lung ventilation occurred, and these findings are consistent with unchanged VO_2_max.

Recently Most et al. [63] reported 29% higher serum ferritin levels in Vit-D sufficient elite male handball and hockey players compared to their Vit-D insufficient counterparts. However, Bacchetta et al. [64] observed a small (10%) but statistically significant decrease in serum ferritin as a result of a single large dose (100,000 IU) of oral Vit-D administration in healthy adults. In our participants, a significant 22% decrease in serum ferritin levels occurred across the 12-week supplementation and RT period, but this was an overall change, not Vit-D supplementation-induced effect. Similarly, other studies have shown that ferritin levels decrease as training program or sports season progresses in male and female soldiers [49,65,66], overweight or obese girls [67] and elite male football players [68]. According to My et al. [68], the decreases in ferritin levels with training load may reflect training tolerance of athletes.

Ferritin is the major iron storage protein [69] and serum ferritin concentration correlates with body iron stores [70]. Serum ferritin levels ≤35 μg/L indicate iron-deficient state, i.e., depletion of iron stores in the bone marrow, liver, and spleen [71]. According to the ferritin criterion, the prevalence of iron deficiency among our participants increased from 15% (6 men) at week 0 to 21% (8 men) at week 12, while the haemoglobin levels remained within the normal range in all men in all time points. Since iron deficiency without anaemia does not affect VO_2_max [71], we consider it unlikely that the low iron status of some participants masked the potential effect of Vit-D supplementation on VO_2_max in our study.

Our second hypothesis that Vit-D supplementation would potentiate the anti-inflammatory effect of RT in Vit-D-deficient young healthy men is supported by the increased IL-10/TNF-α ratio observed in the VD group over the 12-week supplementation and RT period, while there was no such change in the PLC group. IL-10 and TNF-α are considered as a quintessential anti-inflammatory cytokine and prototypical pro-inflammatory cytokine, respectively [72]. As IL-10 reciprocally down-regulates pro-inflammatory cytokine production [72], serum IL-10/TNF-α ratio has been used as an anti-inflammatory index in many previous studies [41,73,74,75]. Recently Silva et al. [41] showed that 12-week RT with elastic bands or on conventional weight machines induced anti-inflammatory effects, including a strong tendency to improve the IL-10/TNF-α ratio and IL-10 levels, in middle-aged and elderly patients with chronic obstructive pulmonary disease (COPD). On the other hand, Nikseresht et al. [75] reported that, considering the magnitude of increases in IL-10/TNF-α ratio, 12-week aerobic interval training had stronger anti-inflammatory effect compared with nonlinear RT in middle-aged obese men. As COPD is an inflammatory disease [76] and overweight and obesity are considered pro-inflammatory conditions [77], the data of Silva et al. [41] and Nikseresht et al. [75] suggest that exercise training may alleviate chronically elevated inflammation in people with health problems. For RT, however, anti-inflammatory effects have been demonstrated also in young healthy normal-weight adults [44,45], although not all studies have led to the same conclusion [78,79]. Of note, none of these research groups [44,45,78,79] controlled Vit-D status of their subjects before or during their participation in RT program. Thus, the findings of our study extend those of other researchers by showing that the anti-inflammatory effect of RT in healthy young normal-weight men may depend on their Vit-D status. Specifically, our data suggest that Vit-D deficiency should be treated to ensure the anti-inflammatory effect of RT. This approach may be important, considering high prevalence of Vit-D deficiency worldwide [80,81] and RT as a potentially effective measure to prevent the onset of chronic inflammation at young age [44,45].

Low-grade inflammation is an important factor involved in the pathogenesis of insulin resistance [82] and chronic Vit-D deficiency parallels with the clinical manifestations of this condition [83]. Six-month Vit-D supplementation in obese adolescents with variable baseline Vit-D status [84] and 12-week RT in obese middle-aged men with uncontrolled Vit-D status [85] significantly reduced HOMA-IR index, i.e., improved insulin resistance. In our participants, the 12-week RT and supplementation period had no impact on fasting serum glucose or insulin levels or HOMA-IR. This finding is consistent with the data from other researchers [22,86] who evaluated the possible combined effect of Vit-D supplementation and RT on insulin resistance.

One of the strengths of the present study is the inclusion of only Vit-D deficient men. This excludes the possible influence of substantial individual differences in baseline Vit-D status on the results. In addition, monitoring participants’ habitual diet three times during the supplementation and RT period enabled us to exclude the possibility that changes in dietary intake influenced the study results. Besides these strengths, our study has some limitations. The increased IL-10/TNF-α ratio observed in the VD group over the 12-week supplementation and RT period revealed the anti-inflammatory effect of Vit-D supplementation, but the effect size was small, there were no changes in other markers of inflammatory state measured, and we did not assess many other known pro- and anti-inflammatory factors. In addition, as whey protein may possess anti-inflammatory effect [87,88], inclusion of a Vit-D only supplemented group in our study would have been appropriate. However, we consider it improbable that post training session whey supplement influenced inflammatory status of participants in our experimental setting because both PLC and VD groups administered whey, but IL-10/TNF-α ratio improved in VD group only.

In conclusion, 12-week Vit-D supplementation increases serum 25(OH)D levels and improves inflammatory status, but has no impact on cardiorespiratory fitness in Vit-D deficient young men engaged in RT.

## Figures and Tables

**Table 1:** Respiratory exchange ratio, peak heart rate, ventilation, and breath frequency during VO_2_max test.

Variables	Placebo (*n* = 17)		Vitamin D (*n* = 20)	
	Week 0	Week 12	Week 0	Week 12
RER	1.09 ± 0.04	1.08 ± 0.05	1.07 ± 0.04	1.10 ± 0.06 *
HR (beats/min)	198.5 ± 8.6	198.0 ± 12.2	196.9 ± 10.7	195.4 ± 11.2
VE (L/min)	145.6 ± 18.6	145.0 ± 19.3	149.0 ± 20.6	148.8 ± 15.4
BF (times/min)	56.2 ± 7.4	56.0 ± 8.6	54.4 ± 6.6	54.7 ± 6.0

Data are presented as mean ± SD. RER, respiratory exchange ratio; HR, heart rate; VE, ventilation; BF, breath frequency. * Statistically significant within-group difference compared to week 0 (*p* < 0.05).

**Table 2:** Serum cytokine levels during 12-week supplementation and resistance training period.

Variables	Placebo (*n* = 18)		Vitamin D (*n* = 21)	
	Week 0	Week 12	Week 0	Week 12
IL-1α (pg/mL)	0.18 ± 0.13	0.22 ± 0.16	0.13 ± 0.07	0.19 ± 0.15
IL-1β (pg/mL)	1.55 ± 1.03	1.52 ± 1.29	1.01 ± 0.45	0.95 ± 0.32
IL-4 (pg/mL)	1.63 ± 0.37	1.67 ± 0.41	1.63 ± 0.35	1.53 ± 0.28
IL-6 (pg/mL)	1.07 ± 1.16	0.82 ± 0.58	0.78 ± 0.62	0.97 ± 0.80
IL-8 (pg/mL)	10.87 ± 3.67	11.06 ± 4.88	12.80 ± 7.12	12.37 ± 9.59
IL-10 (pg/mL)	0.69 ± 0.32	0.76 ± 0.43	0.71 ± 0.49	0.88 ± 0.58
TNF-α (pg/mL)	3.59 ± 0.99	3.53 ± 1.30	3.32 ± 0.87	3.06 ± 1.00
IL-10/TNF-α	0.19 ± 0.07	0.22 ± 0.08	0.23 ± 0.16	0.30 ± 0.19 *
MCP-1 (pg/mL)	171.2 ± 57.4	164.6 ± 54.7	188.8 ± 91.1	172.9 ± 82.6

Data are presented as mean ± SD. IL, interleukin; TNF-α, tumour necrosis factor alpha; MCP-1, monocyte chemoattractant protein 1. * Statistically significant within-group difference compared to week 0 (*p* < 0.05).

**Table 3:** Serum glucose, ferritin, and insulin concentrations and HOMA-IR during 12-week supplementation and resistance training period.

Variables	Group	Week 0	Week 8	Week 12
Glucose (mmol/L)	PLC	5.32 ± 0.28	5.28 ± 0.29	5.48 ± 0.32
	VD	5.12 ± 0.34	5.14 ± 0.38	5.17 ± 0.42
Ferritin (µg/L)	PLC	102.9 ± 72.9	79.5 ± 59.9 *	78.0 ± 59.3 *
	VD	129.6 ± 80.6	107.5 ± 74.5 *	102.1 ± 81.4 *
Insulin (mU/L)	PLC	9.22 ± 3.95	9.46 ± 2.11	9.18 ± 2.20
	VD	7.76 ± 2.26	8.13 ± 3.67	7.41 ± 1.71
HOMA-IR	PLC	2.21 ± 1.04	2.21 ± 0.49	2.25 ± 0.62
	VD	1.78 ± 0.57	1.86 ± 0.90	1.71 ± 0.44

Data are presented as mean ± SD. *n* = 18 in placebo (PLC) and *n* = 21 in vitamin D (VD) group. * Statistically significant within-group difference compared to week 0 (*p* < 0.05).

**Table 4:** Haematological parameters during 12-week supplementation and resistance training period.

Variables	Group	Week 0	Week 8	Week 12
Haemoglobin (g/L)	PLC	155.4 ± 9.9	155.4 ± 10.9	156.4 ± 9.2
	VD	153.0 ± 9.8	151.0 ± 10.8	152.5 ± 10.7
Haematocrit (%)	PLC	46.0 ± 2.5	45.9 ± 2.6	45.8 ± 2.0
	VD	44.7 ± 2.2	44.2 ± 2.2	44.8 ± 2.1
Erythrocytes (×10^12^/L)	PLC	5.26 ± 0.28	5.25 ± 0.27	5.24 ± 0.24
	VD	5.13 ± 0.23	5.08 ± 0.29	5.11 ± 0.28
Leukocytes (×10^9^/L)	PLC	5.8 ± 1.5	5.8 ± 1.4	5.6 ± 1.4
	VD	5.9 ± 1.3	5.8 ± 1.4	6.3 ± 1.9
Thrombocytes (×10^9^/L)	PLC	236 ± 49	242 ± 50	236 ± 39
	VD	237 ± 39	243 ± 41	239 ± 39

Data are presented as mean ± SD; *n* = 18 in placebo (PLC) and *n* = 21 in vitamin D (VD) group.

## Data Availability

The data presented in this study are available on request from the corresponding author (vahur.oopik@ut.ee).
